# Dairy Cows Offered Fresh Chicory Instead of Ensiled Pasture during an Acute Heat Challenge Produced More Milk and Had Lower Body Temperatures

**DOI:** 10.3390/ani13050867

**Published:** 2023-02-27

**Authors:** S. Richard O. Williams, Peter J. Moate, Josie B. Garner, Murray C. Hannah, Khageswor Giri, William J. Wales, Leah C. Marett

**Affiliations:** 1 Agriculture Victoria Research, Ellinbank, VIC 3821, Australia; 2 Centre for Agricultural Innovation, School of Agriculture and Food, Faculty of Veterinary and Agricultural Sciences, The University of Melbourne, Parkville, VIC 3010, Australia; 3 Agriculture Victoria Research, AgriBio, Bundoora, VIC 3083, Australia

**Keywords:** heat stress, cattle, feed type, feed amount

## Abstract

**Simple Summary:**

Dairy cows are particularly susceptible to heat stress, and the type and amount of forage offered could affect their heat load during hot weather. Our aim was to determine the impact of offering dairy cows two different forage types at two different amounts. Cows offered chicory (low fiber) produced more milk and had a lower body temperature than cows that were offered pasture silage (high fiber). Overall, cows that were offered the high amount of forage ate more feed and produced more milk than cows that were offered the low amount, but there was no difference in body temperature between these cows. However, during the heat challenge, cows that were offered the high amount of forage had a greater body temperature than cows that were offered the low amount. This single disadvantage is not sufficient to justify the restriction of feed intake as a strategy for managing hot weather events. While feeding chicory to dairy cows appears to be beneficial, our experiment was not done under commercial conditions, so further work is necessary to confirm our findings.

**Abstract:**

The frequency, duration, and intensity of heat waves in Australia are increasing. To reduce the impact of heat waves on milk production, novel management strategies are required. Altering the forage type and amount offered affect the heat load on dairy cows and offer potential strategies to ameliorate the effects of hot weather. Thirty-two multiparous, lactating Holstein–Friesian cows were assigned one of four dietary treatments: chicory high amount, chicory low amount, pasture silage high amount, or pasture silage low amount. These cows were exposed to a heat wave in controlled-environment chambers. Cows that were offered fresh chicory had similar feed intake to cows that were offered pasture silage (15.3 kg DM/d). However, cows that were offered chicory produced greater energy-corrected milk (21.9 vs. 17.2 kg/d) and had a lower maximum body temperature (39.4 vs. 39.6 °C) than cows that were offered pasture silage overall. Cows that were offered the high amount of forage had greater feed intake (16.5 vs. 14.1 kg DM/d) and energy corrected milk yield (20.0 vs. 17.9 kg/d) than cows that were offered the low amount, as intended, but with no difference in maximum body temperature (39.5 °C). We conclude that feeding chicory instead of pasture silage to dairy cows shows promise as a dietary strategy to ameliorate the effect of heat exposure, and there was no advantage in restricting feed amount.

## 1. Introduction

The frequency, duration, and intensity of heat waves in Australia are increasing [1]. Dairy cows are particularly susceptible to heat stress, [2] with both short- and long-term effects on reproduction, health [3], and milk production [4]. Overall, heat production as a result of ingesting feed is a combination of heat that is generated during fermentation in the rumen, energy expenditure during digestive processes and the metabolism of digestion end-products [5]. Metabolic heat is influenced by the chemical nature of the end products of ruminal fermentation and digestion, which is in turn determined by the types and amounts of the different feeds consumed. For example, dietary fat does not ferment in the rumen and can therefore be expected to not produce fermentation heat. In contrast, starch and other readily fermentable carbohydrates produce heat in the rumen [6,7]. Furthermore, fibrous feeds that ferment in the rumen to produce acetate result in greater metabolic heat production than feeds that ferment to produce propionate, as the metabolism of acetate generates more heat than propionate [8].

The fiber within a feed comprises its structural carbohydrates, and the fiber concentration in a feed has been linked to differences in voluntary feed intake between forage types [9]. There is evidence that feeding cows a low-fiber diet (≤30% neutral detergent fiber, NDF) during heat stress can have a positive effect on milk production compared with feeding cows a high-fiber diet (42% NDF) [10]. It has also been observed that cows fed high-fiber (NDF) diets during hot weather consume less feed than cows offered low-fiber diets [11]. Additionally, Miron et al. [12] demonstrated that reducing the NDF concentration of the diet resulted in increased milk production and dry matter intake (DMI), and decreased symptoms of heat stress. Offering a highly digestible (low NDF) and palatable forage type to cows during periods of hot weather may improve voluntary feed intake and milk production. During the summer months, dryland dairy farming in southern Australia relies heavily on pasture silage. Chicory (*Cichorium intybus* L.) has a lower concentration of NDF in comparison to perennial ryegrass (*Lolium perenne* L.) [13,14]. The high dry matter digestibility and fast rumen passage rate of chicory may provide cows with the opportunity to increase their voluntary feed intake [15]. Cows grazing on chicory have been shown to produce more milk and milk solids than cows grazing on perennial ryegrass swards during summer and autumn [13,15,16]. Minnee et al. [17] found that the inclusion of chicory increased milk yields by 2.7 kg/day compared to when feeding on perennial ryegrass alone, and that when the metabolizable energy (ME) of perennial ryegrass was low (9.6 MJ/kg of DM) compared to chicory (12.3 MJ/kg of DM) as the proportion of chicory in the diet was increased to 40%, milk yield increased from 9.9 to 12.6 kg/cow per day. In addition, when the ME of the perennial ryegrass was greater (10.5 MJ/kg of DM) milk yield increased from 14.9 to 15.7 kg/cow per day. It is possible that feeding a large amount of chicory will result in a decline in milk fat concentration due to the low NDF concentration. However, there are studies in which chicory was fed at up to 15 kg of DM or 60% of the diet, with no negative effect on milk composition [17,18]. In comparison to ryegrass silage, chicory is a suitable alternative forage for high-producing dairy cows during summer because it has lower NDF concentration and greater DM digestibility. The low structural carbohydrate concentration of chicory has the potential to ameliorate the increase in body temperature through reduced fermentation heat. This may improve milk yield responses during heat stress compared to when pasture silage is fed to dairy cows.

The amount of feed offered during hot weather may be more critical to total heat production than the fiber concentration [10]. For example, the heat production of beef heifers managed on a high feed intake was greater than those managed on a low feed intake [19]. The relationship between intake of ME and heat production has been suggested to be curvilinear [20]. While the feed intake of dairy cows has been reported to decline with increasing dietary NDF, the rate of decline is lower in hot weather [10]. This suggests that there is potential to reduce heat stress responses by altering the forage type and amount offered to lactating dairy cows during a period of increased risk of heat stress.

The aim of this experiment was to determine the impact of offering dairy cows a diet containing forages of different proportions of structural carbohydrates on the DMI, milk production and physiological responses in a controlled heat challenge. As it is common practice to feed pasture silage to dairy cows during summer as the main forage source, this was selected as the forage to compare with chicory, a forage with considerably lower structural carbohydrate concentration. We hypothesized that (1) cows offered fresh chicory would have greater feed intake and greater energy-corrected milk (ECM) but lower body temperature compared to cows offered pasture silage; (2) cows offered a high amount of forage would have greater feed intake, ECM and body temperature compared to cows offered a low amount of forage; and (3) during the heat challenge, the decrease in DMI and ECM of cows offered fresh chicory would be less than that of cows offered pasture silage.

## 2. Materials and Methods

### 2.1. Cows and Diets

Thirty-two multiparous, lactating Holstein–Friesian cows producing 28.9 ± 3.36 kg milk/d (mean ± standard deviation) with 582 ± 46.8 kg body weight (BW), 147 ± 26.8 days in milk (DIM), 5.0 ± 1.32 years of age, 4.5 ± 0.17 body condition (8 point scale; 1 = skinny, 8 = fat; [21]) and 100 ± 3.2 heat tolerance breeding value (DataGene, Bundoora, Victoria, Australia; 100 = national breed mean) were assigned one of four treatment diets.

The 4 treatment diets were (1) CH-H—5 kg DM grain mix plus 13 kg DM of fresh cut chicory, (2) CH-L—5 kg grain mix plus 10 kg DM of fresh cut chicory, (3) PS-H—5 kg DM grain mix plus 13 kg DM pasture silage (predominantly perennial ryegrass), or (4) PS-L—5 kg DM grain mix plus 10 kg DM pasture silage. The grain mix consisted of cracked wheat grain (*Triticum aestivum* L.) (815 g/kg DM), cracked lupins (*Lupinus albus* L.) (112 g/kg DM), minerals (51 g/kg DM), E Mag 523 (6 g/kg DM, Queensland Magnesia Pty Ltd., Toowong, Queensland, Australia), and limestone (16 g/kg DM). During the recovery period, the CH-H diet consisted of the grain mix plus ad libitum fresh cut chicory, and the PS-H diet consisted of the grain mix plus ad libitum pasture silage. The CH-L and PS-L diets remained unchanged. Water was available ad libitum at all times except during milking.

Compositions of the main dietary ingredients are shown in Table 1. Cows were offered their grain mix during milking and were allowed up to 30 min to consume the grain mix. After this time, refusals were collected, cows were moved to the animal house (described later), and the forage was offered.

Chicory was freshly cut at 05:00 and 13:30 at a height of 5 cm above ground using a front-mount mower (Pöttinger NovaCat 306F; Alois Pöttinger Maschinenfabrik GmbH, Grieskirchen, Austria) and loader wagon (Quantum 3500P; Claas GmbH & Co., Harsewinkel, Germany) mounted on a tractor (Arion 530; Claas GmbH & Co., Harsewinkel, Germany).

Silage was prepared once per day in the early afternoon. Silage was collected from the fresh face of a bunk and placed into a mixer wagon (Jaylor 5100 Trailer; Jaylor, East Garafraxa, ON, Canada) for homogenization. Quantities for individual cow feeds were weighed into plastic boxes of 50 L. Boxes of the afternoon feed were set aside until required. Boxes of the morning feed were stored at 4 °C until required.

### 2.2. Experiment Design

The experiment had two treatment factors, each with two levels—forage types (chicory vs. silage) and forage amounts (high vs. low), resulting in a 2 × 2 factorial structure.

Although 32 cows were enrolled in the experiment, only 30 places were available in the controlled-environment chambers. The 4 dietary treatments (CH-H, CH-L, PS-H and PS-L) were assigned in a 5-row by 6-column design (Table 2) in which each row corresponded to a cohort, each column to a chamber, and the cells to individual cows.

The treatment allocation was randomized according to the row-column design, by permutation of rows and permutation of columns. Provision for the possibility of problems due to animal health or behavior was made by starting cows in two batches 8 days apart. Cows in the same treatment from the next block were used to replace cows removed due to animal health and behavior. Cows that recovered were used in a subsequent block in the position of the same treatment. Block 5 was adjusted by choosing 6 cows from the remaining 8 to boost the number of animals in treatments where previously cows had failed to complete the heat challenge or had been removed for animal health or behavior issues. Each cohort of 6 cows contained all 4 dietary treatments plus two dietary treatments represented twice. The doubled-up treatments were selected in a way to give near-balance of treatments over the 5 cohorts. The 5 cohorts correspond to 5 calving-date blocks, cows were similar within cohort in their DIM, and treatment groups were balanced (in terms of mean and standard deviation) for cow body weight, 7 d milk yield, lactation number, balanced performance index (DataGene, Bundoora, VIC, Australia) and heat-tolerance breeding value. This was achieved using covariate design [22] implemented in GenStat 19 (VSN International Ltd., Hemel Hempstead, UK).

During the covariate period (days 1 to 4), cows were managed as a single group in a paddock at ambient conditions with measurements made of milk yield and composition and of BW. During this time, cows continued to be offered the same diet as the main research herd: 7 kg DM/cow/d of a grain mix (cracked barley grain 500 g/kg DM, solvent-extracted canola meal 260 g/kg DM, and cracked wheat grain 240 g/kg DM) during milking and about 9 kg DM/cow of ryegrass pasture and 5 kg DM/cow of pasture silage per day in a paddock.

The adaptation to diet commenced on day 5. Cows were moved to the experiment facilities, where they were offered their forage in individual feed stalls for 2 periods of 3.5 h in a well-ventilated animal house [23] and rested on a 560 m^2^, roofed, rubber-floored loafing pad adjacent to the feed stalls. All cows were transitioned to the high-amount version of their treatment diet over 3 days (days 5 to 7), and adapted to the high-amount version of their diet for 7 days (days 8 to 14); then, cows on the low-amount treatments had their feed-offer reduced to the low amount for the remainder of the experiment. Days 19 to 21 were taken as the base period.

Cows were moved to controlled-climate chambers [24] for the pre-challenge period on day 22, where measurements were made at thermoneutral conditions.

The heat challenge was then conducted for 2 days (days 23 to 24). From 06:01 to 12:00, the set conditions were 30 °C and 50% RH (temperature humidity index, THI = 80.1); from 12:01 to 18:00, setpoints were 33 °C and 50%RH (THI = 84.2); and from 18:01 to 06:00, the setpoints were 26.0 °C and 60%RH (THI = 74.5).

The recovery period was monitored under ambient conditions for 4 d (days 25 to 28). Cows on the high-amount diets were offered ad libitum forage during this period.

**Figure 1:**
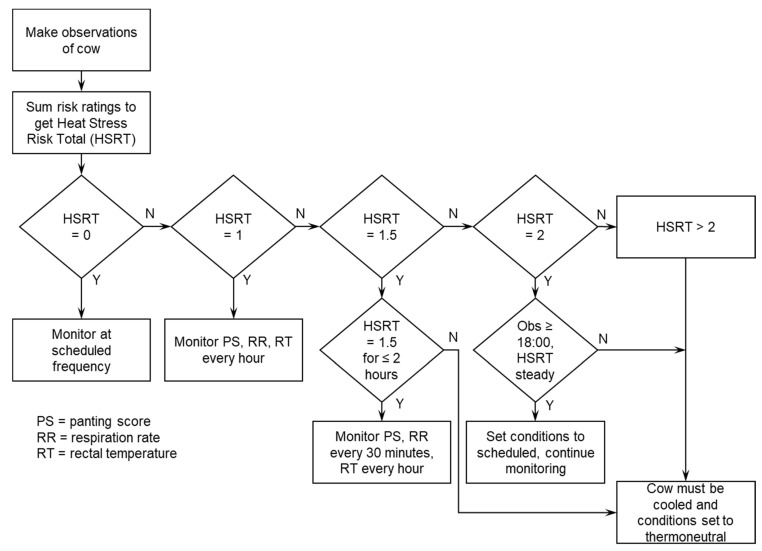
Flowchart of actions taken depending on the heat-stress risk total (HSRT) of the cow.

To ensure animal welfare and health were not compromised during the experiment, the welfare status of individual cows was monitored using a novel heat-stress risk approach. The aim of this was to objectively identify the point where animals were at risk of not being able to recover from the heat challenge and to signal removal from the experiment to maintain animal health and welfare. Each cow was individually assessed for panting score, respiration rate, and rectal temperature multiple times per day during the heat challenge period. A panting score was assigned for each cow based on the scale of Gaughan et al. [25]. To obtain respiration rate, cows were visually observed, and the number of breaths (flank movements) counted in a 20 s period were multiplied by three. Rectal temperature was measured at milking (06:00 h and 15:00 h) during the heat challenge period using a large animal thermometer (AG-Medix, LLC Animal Thermometer AG-102 R01, Wisconsin, USA). Value ranges of respiration rate, panting score and rectal temperature were then each assigned a heat-stress risk rating (Table 3) based on scientific thresholds reported in the literature [25] and our previous observations during experiments conducted under similar conditions [26,27]. The individual cows’ risk ratings were then added together to give a heat-stress risk total (HSRT). Based on the cow’s HSRT, additional monitoring actions were implemented to maintain animal welfare and health. These actions are detailed in Figure 1.

If necessary, cows were cooled by opening the chamber doors and setting the conditions to thermoneutral (17 °C, 60% RH). Chamber doors were closed once the cow rectal temperature was below 41.0 °C or when conditions within the chamber were cooler than ambient, whichever occurred first, and cows were monitored using the heat-stress risk approach. Cooled cows stayed within their chamber at thermoneutral conditions for the remainder of their scheduled time in the chamber. Cows that were cooled had their recovery monitored from the day that chamber temperature was reduced.

### 2.3. Feed and Feed Analysis

Feed was offered in two equal portions immediately following the morning and afternoon milkings. Feed refusals were collected and weighed after each feed. Dry matter concentration was determined on representative samples of each grain, and minerals were collected every Tuesday and Wednesday of the experiment, as well as on representative samples of chicory collected at every feeding, and samples of silage were collected every afternoon. These feed samples were dried in a forced draft oven at 105 °C for 24 h.

Samples of the grains, chicory, and silage offered were collected daily; a 100 g sub-sample was taken and added to the weekly (Monday to Sunday) bulk sample for each feed type and stored at −18 °C. Bulked samples were subsequently oven-dried at 64 °C for 48 h, ground to pass through a 1.0 mm screen (MEP rotor mill; Retsch GmbH, Haan, Germany), and then analyzed for crude protein (CP), soluble protein, acid detergent fiber (ADF), neutral detergent fiber (NDF), lignin, non-fiber carbohydrate, starch, ash, total digestible nutrients (TDN), crude fat (ether extract, EE), sodium, potassium, calcium, magnesium, phosphorous, sulfur, and chloride by traditional chemical analytical methods according to the procedures of Dairy One [28]. Metabolizable energy was calculated using Equation (1) [29].

ME (MJ/kg) = 14.55 − 0.0155 × ADF (g/kg)(1)

### 2.4. Milk Production and Composition

Cows were milked twice daily, at ~06:00 h and ~15:00 h. Throughout the experiment, milk yield was measured for each cow at each milking. When cows were not in the chambers, milk yield measurements were recorded using a DeLaval Alpro milk metering system (MM27BC; DeLaval International, Tumba, Sweden), and milk samples for composition analysis were collected during the covariate, pre-challenge, heat challenge, and recovery periods. When cows were in the controlled-environment chambers, milk yield measurements were made by collecting and weighing the milk from individual cows. Milk samples were analyzed for fat, protein, and lactose using a mid-infrared milk analyzer (Bentley FTS, Bentley Instruments, Chaska, MN, USA).

Energy-corrected milk standardized to 4.0% fat and 3.3% protein was calculated using Equation (2) [30]:

ECM (kg/d) = (milk yield (kg) × (376 × fat% + 209 × protein% + 948))/3138,(2)

Milk yields were assigned such that milk from a PM milking and the following AM milking were matched to feed intake and weather conditions from the day of the PM milking.

### 2.5. Physiology

Body temperature was measured intravaginally (vaginal temperature) and recorded every 5 min during the covariate, pre-challenge, heat challenge, and recovery periods using temperature loggers (iButton DS1922L; Maxim Integrated, San Jose, CA, USA) as described by Garner et al. [24] and set to high-resolution mode.

Skin temperature was measured every day during the pre-challenge, heat challenge, and recovery periods at approximately 06:00 h and 15:00 h. The surface temperature of the cow was measured using a non-contact infra-red thermometer (Oricom HFS1000; Oricom International, South Windsor, NSW, Australia) in object temperature mode at each of the left paralumbar fossa (left flank), the left trapezius muscle (neck) outside of the left front leg at the mid-point of the radius (leg upper), and the outside of the left front leg at the mid-point of the metacarpus (leg lower).

Using the same methods described in the HSRT, respiration rate and panting score were measured by visually observing each cow at approximately 05:45 h and 14:45 h every day during the pre-challenge, heat challenge, and recovery periods. An additional observation was made at 11:45 h on each day of the 2-day heat challenge. Rectal temperature was measured at 06:00 h and 15:00 h during the heat challenge period.

### 2.6. Blood

Blood samples were collected from each cow at approximately 14:45 h via coccygeal venipuncture during the pre-challenge period (day 22), on day two of the heat challenge period (day 24) and once during the recovery period (day 26). On each occasion, three 10 mL blood samples were collected—one into a vacutainer containing potassium EDTA for plasma collection and two samples into a vacutainer containing clotting activators for serum collection (BD Vacutainer System, Plymouth, UK).

Using a syringe, approximately 0.1 mL of blood from the EDTA tube was removed immediately after collection and inserted into the reader chip of an auto-calibrated, portable, blood gas analyzer (Epoc Host2 Zebra MC55A0, Epocal Inc. Ottawa, ON, Canada), as per manufacturer’s instructions. Analytical variables determined were blood pH, partial pressure of CO_2_ (pCO_2_), partial pressure of O_2_ (pO2), Na^+^, K^+^, Ca^++^, Cl^−^, glucose, lactate, creatinine, and hematocrit (Hct); the calculated values of bicarbonate (cHCO_3_^−^), total CO_2_ (cTCO_2_), base excess of extracellular fluid, base excess of blood, hemoglobin (cHgb), and oxygen saturation (cSO_2_); and anion gap K^+^ (AGapK).

The remaining sample was then placed on ice before centrifugation within 30 min of collection at 1500× *g* and 4 °C for 10 min. Plasma was decanted into storage vials and stored at −20 °C until analysis. The two samples for serum collection were kept at 25 °C for 1.5 h before centrifugation at 1300 × *g* and 25 °C for 10 min. Serum was decanted into storage vials and stored at −20 °C until analysis. A subset of serum samples was transported on ice to Regional Laboratory Services (Benalla, VIC, Australia) within 24 h of collection. Samples were analyzed for concentrations of beta-hydroxy butyrate (BHB), non-esterified fatty acids (NEFA), urea total protein, albumin, glucose, haptoglobin, and phosphorus using a Kone 20 XT clinical chemistry analyzer (Thermo Fisher Scientific, Waltham, MA, USA), with reagents supplied by Randox Laboratories (Crumlin, UK) for fatty acids, and BUN and Regional Laboratory Services (Benalla, VIC, Australia) for BHB, albumin, and total protein. Haptoglobin concentration was measured using a colorimetric rate assay [31].

### 2.7. Calculations and Statistical Analyses

The THI was calculated using Equation (3) [32]

THI = Tdb + (0.36 × Tdp) + 41.2,(3)

where:

Tdb = hourly dry bulb temperature (°C);
Tdp = dew point temperature (°C);
RH = relative humidity (%);
b = [log_10_(RH/100.0) + (17.27 × Tdb)/(237.3 + Tdb)]/17.27;
Tdp = (237.3 × b)/(1.0 − b).

Temperature and relative humidity data were collected every 1 min during the pre-challenge and recovery periods using Minnow 1.0 data loggers (Senonics LLC, Arvada, CO, USA) positioned 2 m above the ground on the fence line between the feed stalls and the loafing area. During the heat challenge, temperature and relative humidity data were recorded every 1 min by the control system of the controlled-climate chambers. A THI threshold of 68 was used to determine if weather conditions were imposing heat stress on the animals as this is the point that has previously been identified as being when weather conditions start affecting milk production [33].

Duration of vaginal temperature greater than 38.8 °C was calculated as the number of minutes that vaginal temperature exceeded the 38.8 °C threshold on a given day (06:00 h to 05:59 h the next calendar day). The threshold of 38.8 °C was used because this was the mean vaginal temperature of cows during the thermoneutral period in the experiment of Garner et al. [24].

Only data from cows that completed the heat challenge period were used in the analysis of treatment effects. Data from 27 cows (CH-H, n = 7; CH-L, n = 7; PS-H, n = 7; PS-L, n = 6) were used in the following statistical analyses.

The effect of heat challenge (trend over time) on milk production variables, body (i.e., vaginal) temperature, and respiration rate were analyzed by examining the linear regression slopes for mean changes between pre-challenge and heat challenge periods, heat challenge and recovery periods, and pre-challenge and recovery periods. These response variables were averaged across the treatments for each cohort, and this data was used for the t-test to examine if the slope was significantly different from zero at a 5% level of significance. The cohort was used as a replicate since it provided independent replication with respect to time.

Given our experiment design, examining the main effect of forage type involved a comparison between levels of forage type only, and examining the main effect of forage amount involved comparison between levels of forage amount only. The interaction examined how effect of forage type changes with the levels of forage amount.

The effects of treatments (feed type, feed amount) and their interaction (feed type × feed amount) on response variables in each period and between any two periods were analyzed using Linear Mixed Models (LMM), with individual cows as the unit of analyses. The fixed effects included the main effect of feed type, the main effect of feed amount, and their interaction. The effect of covariate (same variable measured in covariate period if available) and effect of cohort were also fitted as fixed effects. The effect of cow was fitted as random effect and was used as a residual term. The response variables were constructed to address the hypotheses, with one datum per animal by averaging per animal in each period. Also, a weighted mean per animal over the 10 days of measurement (3-day base period, 1-day pre-challenge period, 2-day heat challenge period and 4-day recovery period) for milk production was constructed. The change variables were calculated for each individual cow by taking the difference between the mean value of a specific variable measured during the pre-challenge period and the value of the same variable measured during the heat-challenge period. Similar change variables were calculated between the first and second days of recovery (initial recovery). The Linear Mixed Models (LMM) used to analyze these variables can be written in the following form:

$$y=\mu +\beta y_{c}+K+F+A+F.A+\varepsilon$$

where *y* is the response variable of interest, *µ* is an overall constant (grand mean), $y_{c}$ is the covariate (same variable if available from the covariate period), β is the linear coefficient, *K* is the effect of cohort, *F* is the main effect of feed type, *A* is the main effect of feed amount, *F.A* is the interaction between feed type and feed amount, and ε is the random error for an individual cow assumed to follow normal distribution with zero mean and constant variance. The LMM were fitted using a restricted maximum likelihood (REML) algorithm in GenStat. Histograms of residuals and plots of residuals versus fitted values were examined for normality of distribution with constant variance. Three cows that did not complete the heat challenge were excluded from the analyses. All statistical analyses were undertaken using GenStat (GenStat release 21, VSN International Ltd., Hemel Hempstead, UK).

Any *p*-values of < 0.05 were considered significant, and those ≥0.05 and <0.10 were considered a trend. The Duncan’s letters indicating significant differences amongst the comparisons of treatment means were based on Fisher’s unprotected Least Significant Difference (LSD) test. When Fisher’s unprotected LSD is used, the comparisons are tested even when the *p*-value for the term generating these means is not statistically significant.

## 3. Results

**Figure 2:**
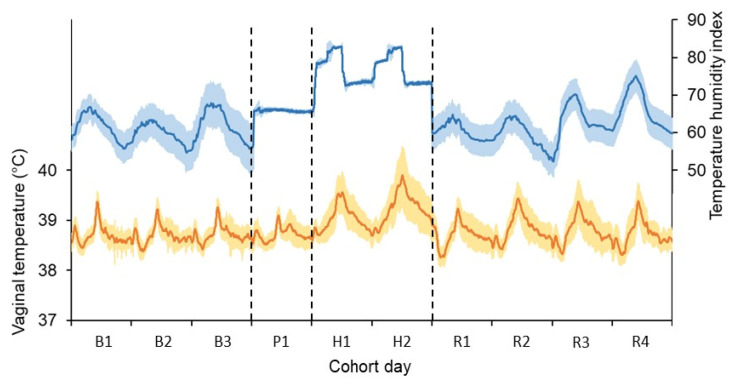
Mean environmental conditions experienced by the cows (blue line) and mean vaginal temperature of all cows (orange line) during the base period (B), pre-challenge (P), heat challenge (H), and recovery period (R). Shading bands show ± one standard deviation from the mean. The pre-challenge and heat challenge were generated in controlled-climate chambers. Cows were in ambient conditions at all other times.

Weather conditions during the base period (ambient conditions) included an air temperature of 18.4 ± 3.97 °C (daily mean ± standard deviation), relative humidity of 79 ± 14.5%, and THI of 66 ± 5.1. During the pre-challenge (in chambers) period, the cows experienced an air temperature of 18.8 ± 0.44 °C, relative humidity of 68 ± 2.1%, and THI of 66 ± 0.6. During the heat challenge (in chambers) period, the cows experienced an air temperature of 26.5 ± 3.15 °C, relative humidity of 67 ± 5.0%, and THI of 76 ± 4.1. Weather during the recovery period (ambient conditions) included an air temperature of 17.0 ± 4.25 °C, relative humidity of 79 ± 15.0%, and THI of 64 ± 5.5. The daily pattern of THI experienced by the cows during the base period, pre-challenge, heat challenge and recovery periods is shown in Figure 2.

The coefficient of variation in THI was 0.01 during the pre-challenge period, 0.05 during the heat challenge, and 0.09 during the recovery period.

### 3.1. Effect of Heat Challenge

**Figure 3:**
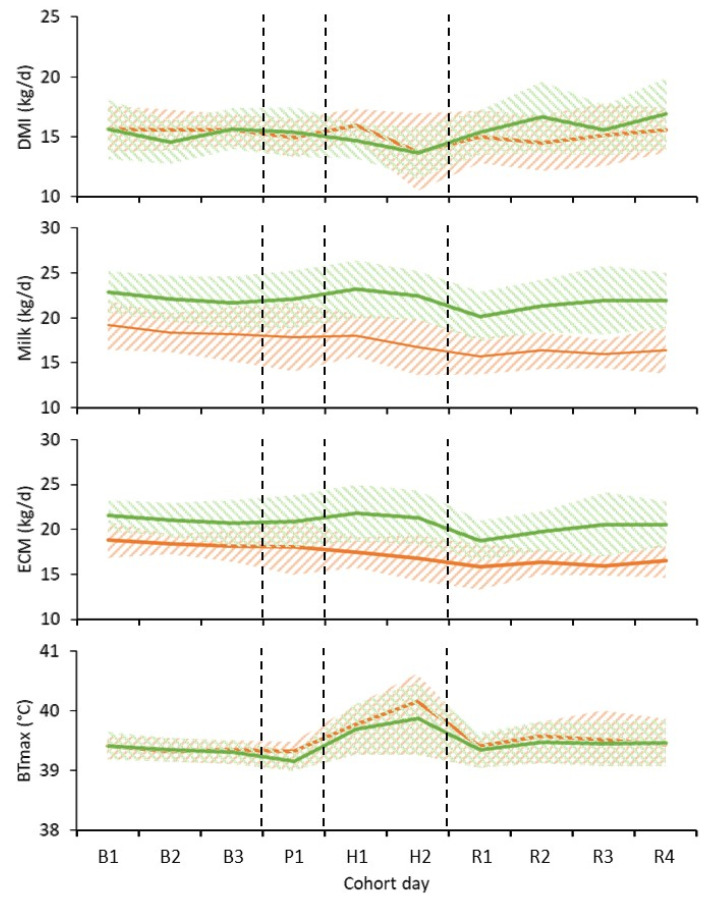
Mean daily dry matter intake (DMI), milk yield (Milk), energy-corrected milk yield (ECM) and maximum vaginal temperature (BTmax) of cows offered chicory (green line) or pasture silage (orange line) during the base period (B), pre-challenge (P), heat challenge (H), and recovery period (P). Each band shows ± one standard deviation from the forage-type mean. The pre-challenge and heat challenge were generated in controlled-climate chambers. Cows were in ambient conditions at other times.

Daily changes in DMI, milk yield, ECM and maximum vaginal temperature during the pre-challenge, heat challenge and recovery periods are shown in Figure 3. The heat challenge induced heat stress symptoms in all cows (Table 4). Mean vaginal temperature and respiration rate were greater in all cows during the heat challenge compared to during other periods (*p* < 0.05). While there was no effect of the heat challenge on feed or on milk yield during the heat challenge, we note that minimum milk production across the 10 days of measurements occurred on the day immediately after the heat challenge.

### 3.2. Challenge Completion

In response to adjusting cohort 5 to balance the cows completing the heat challenge, the two cows not subjected to the heat challenge included one on the CH-L diet and one on the PS-L diet. One cow (PS-H) scheduled for the heat challenge became ill prior to the heat challenge, and two cows (1 CH-H, 1 PS-L) had their heat challenge cut short because their heat-stress risk total exceeded the pre-determined threshold.

### 3.3. Dry Matter Intake

Feed intake across the 10 days of measurements, inclusive of the pre-challenge, heat challenge, and recovery periods (Table 5), was not affected by feed type, but as intended, cows offered more feed ate more (*p* < 0.001). The metabolizable energy of the chicory was greater than the pasture silage. Thus, metabolizable energy intake (MEI) was greater in cows offered chicory than those offered pasture silage (*p* = 0.020). Mean amount of grain refused per cow per day across the entire experiment was 25 g DM, but this measure ranged from 0 to 2.5 kg DM/day for individual cows.

During the pre-challenge period, DMI (Table 5) was not affected by feed type, but the cows offered the high amount of feed ate more than those cows offered the low amount (*p* < 0.001). Intake of ME (Table 5) was greater in cows offered chicory than those offered pasture silage (*p* = 0.019) and MEI was greater in cows offered the high amount of DM than in those offered the low amount of DM (*p* < 0.001).

During the heat challenge, DMI was not affected by feed type, but cows offered the high amount of forage ate more than those offered the low amount (*p* = 0.008). Intake of ME was not affected by feed type (*p* = 0.489), but MEI was greater in cows offered the high amount of feed than those cows offered the low amount (*p* = 0.021).

From the pre-challenge period to the heat challenge, DMI and MEI declined, and there was no effect of feed type or feed amount.

During the initial recovery, DMI increased for cows offered chicory but continued to decline for cows offered pasture silage (*p* = 0.022). There was an interaction between feed type and amount for DMI during the initial recovery period such that DMI increased for cows offered the high amount of forage but not for those offered the low amount (*p* = 0.017). This reflects all cows on the high amount of forage being offered more during the recovery period, but only the cows offered the CH-H diet consumed this extra feed (Table 5). The intake of ME increased during the initial recovery period for cows offered chicory, but continued to decline for cows offered pasture silage (*p* = 0.031). There was an interaction between feed type and amount for DMI during the initial recovery period such that MEI increased for cows offered the high amount of feed but not for those offered the low amount (*p* = 0.013), reflecting the change in intake. Again, this reflects the cows offered the CH-H diet consuming more feed than the other cows.

### 3.4. Milk Yield

Across the 10 days of measurements, cows offered chicory had greater yields of milk (*p* < 0.001), ECM (*p* < 0.001), fat (*p* = 0.017), protein (*p* < 0.001), and lactose (*p* < 0.001) than cows offered pasture silage (Table 6). The concentration of fat in their milk was lower (*p* < 0.001), and the concentration of protein was greater (*p* < 0.001) for cows offered chicory compared to cows offered pasture silage. Cows offered the high amount of feed produced more milk than cows offered the low amount (*p* < 0.004) but there was no effect on milk composition. There were interactions between type and amount of forage for milk yield (*p* = 0.003), ECM yield (*p* < 0.001), fat yield (*p* = 0.035), and protein yield (*p* = 0.021).

During the pre-challenge period, milk yields (*p* = 0.002) and yields of ECM (*p* = 0.021) and milk protein (*p* < 0.001) were greater from cows offered chicory than from cows offered pasture silage, and there was no effect of feed type on milk fat yield. Compared to cows offered chicory, cows offered pasture silage had a greater concentration of milk fat (*p* = 0.003) but a lower concentration of milk protein (*p* = 0.017). There was no effect of feed amount on milk parameters. There was an interaction between forage amount and type on milk yield (*p* = 0.038).

During the heat challenge, cows offered chicory had greater yields of milk (*p* < 0.001), ECM (*p* < 0.001), milk fat (*p* = 0.005), and milk protein (*p* < 0.001) than cows offered pasture silage. Cows offered chicory had a lower concentration of milk fat (*p* = 0.001) but a greater concentration of milk protein (*p* = 0.004) than cows offered pasture silage. Cows offered the high amount of feed had greater yields of milk (*p* = 0.006), ECM (*p* < 0.001), fat (*p* = 0.004), and protein (*p* = 0.001) than cows offered the low amount. There were interactions between type and amount of forage for milk yield (*p* = 0.014), ECM yield (*p* < 0.001), and fat yield (*p* = 0.003).

From the pre-challenge period to the heat challenge, feed type had no effect on the change in milk parameters. Feed amount affected the change in yields of milk and milk components (*p* < 0.05). Cows offered the high amount of feed had a positive change in yields of milk, ECM, milk fat, and milk protein, whereas this change was negative for cows offered the low amount of feed (Table 6). There were no interactions.

During the initial recovery, the only difference observed in milk parameters was that the increase in the yield of milk protein was greater for cows offered chicory than those offered pasture silage (*p* = 0.046). There were no interactions.

### 3.5. Body Temperature

Across the 10 days of measurements, cows offered chicory had lower mean (*p* = 0.022) and maximum (*p* = 0.024) vaginal temperatures (Table 7) than cows offered pasture silage. Feed amount had no effect on vaginal temperature parameters.

During the pre-challenge period, all vaginal temperature parameters were lower in cows offered chicory than in cows offered pasture silage (*p* < 0.006). There was no effect of feed amount on vaginal temperature parameters.

During the heat challenge, cows offered chicory had a lower mean vaginal temperature (*p* = 0.048) and duration above 38.8 °C (*p* = 0.010) than cows offered pasture silage. They also tended to have a lower maximum vaginal temperature (*p* = 0.059) than cows offered pasture silage. Cows offered the high amount of feed had a greater maximum vaginal temperature (*p* = 0.011) and tended to have a greater mean vaginal temperature (*p* = 0.059) than cows offered the low amount.

From the pre-challenge to the heat challenge, feed type had no effect on change in vaginal temperature parameters (Table 7). Cows offered the high amount of feed tended to have a greater increase in vaginal temperature (*p* = 0.069) than cows offered the low amount, and they did have a greater increase in maximum temperature (*p* = 0.006).

During the initial recovery, cows offered chicory tended to have a lower change in mean vaginal temperature (*p* = 0.085) than cows offered pasture silage. There was no effect of feed amount on change in any vaginal temperature parameters.

### 3.6. Respiration Rate and Skin Temperature

During the pre-challenge period, respiration rate (*p* = 0.063) tended to be greater, but left flank temperature (*p* = 0.092) and leg-upper temperature (*p* = 0.086) tended to be lower in cows offered chicory compared to those offered pasture silage (Table 8). Leg upper (*p* < 0.001) and lower (*p* = 0.014) temperatures were greater in cows fed the high amount of feed than in those fed the low amount.

During the heat challenge, cows offered chicory tended to have a lower upper-leg temperature (*p* = 0.097) than cows offered pasture silage. Cows offered the high amount of feed had greater respiration rates (*p* = 0.035) and skin temperatures (*p* < 0.016) than cows offered the low amount.

### 3.7. Blood Analytes

Select blood analytes are presented in Table 9, and a complete listing of blood analytes measured is available in Table S1 Blood parameters.

During the pre-challenge period, glucose concentration tended to be lower (*p* = 0.062) in cows offered chicory compared to those offered pasture silage. Cows offered the high amount tended to have greater blood pH (*p* = 0.069) than those offered the low amount.

During the heat-challenge, cows offered chicory had lower blood concentrations of BHB (*p* = 0.006) and glucose (*p* = 0.024) than cows offered pasture silage. Cows offered the high amount tended to have a lower concentration of haptoglobin (*p* = 0.090) than cows offered the low amount.

## 4. Discussion

### 4.1. Forage Type

The cows that were offered fresh chicory had similar feed intake compared to the cows that were offered pasture silage during each period of the experiment, except for during the recovery period. However, the cows that were offered chicory produced more ECM and had a lower body temperature than the cows that were offered pasture silage. Thus, we can only accept the ECM and body temperature portions of our first hypothesis and reject the intake portion. During the recovery period, cows on the high feed amount were offered a greater quantity of feed, but only the cows that were offered chicory chose to consume this extra feed. This greater voluntary intake of feed may be explained by the lower body temperature experienced by the cows that were offered chicory [34] or the higher dry matter digestibility and faster rumen passage rate of chicory [15] compared to pasture silage. Also, we speculate that the lower dry matter concentration of the chicory may have resulted in greater palatability compared to the pasture silage.

The energy corrected milk yield from the cows that were offered chicory was greater than that from the cows that were offered pasture silage during all periods of the experiment, including the heat challenge period. This response to feeding chicory is in agreement with previous reports wherein chicory was fed in place of fresh ryegrass [13,15,16]. The ECM our cows that were fed chicory was 3.6 kg greater than our cows that were fed pasture silage, and this difference is mostly accounted for by the difference in intake of ME (13 MJ = 2.6 kg ECM, [29]). The greater ECM yield from the cows that were offered chicory compared to those that were offered pasture silage was driven by a greater milk yield and a greater concentration of milk protein, despite the concentration of milk fat being lower in milk from the cows that were fed chicory than those fed pasture silage. This lower milk fat concentration in the cows that were offered chicory is in contrast to previous reports wherein feeding chicory had no effect on the concentration of milk fat [17,18]. In those studies, chicory was no more than 60% of the total diet, while the cows in our experiment were offered chicory at 73% of total DMI. The lower concentration of milk fat is thought to be due to the chicory diet having a lower concentration of NDF than the pasture silage diet, since the proportion of fiber in the diet has previously been correlated with the concentration of fat in milk [35].

Vaginal temperature was lower over the 10 days of measurement in the cows that were offered chicory than in the cows offered pasture silage. This is consistent with the heat increment of the chicory being lower than that of pasture silage due to the lower concentration of fiber [10]. While the increase in body temperature induced by the heat challenge was not affected by diet type, the cows that were offered chicory had a lower temperature during the pre-challenge period than the cows that were offered the pasture silage, thus they also had a lower body temperature during the heat challenge. This suggests that while feeding chicory may not affect the change in body temperature due to a heat challenge, the lower body temperatures during the heat challenge means that our cows that were fed chicory experienced less thermal stress than those that were offered pasture silage. This is supported by the fact that the chicory-fed cows had a lower body temperature but had a similar respiration rate and skin temperature to the cows fed pasture silage. It is plausible that the tendency for greater respiration rate of the cows that were offered chicory contributed to a lower body temperature compared to cows offered pasture silage.

### 4.2. Forage Amount

The cows that were offered the high amount of forage had a greater DMI and ECM yield than the cows offered the low amount, as intended. However, there was no overall difference in body temperature for the 10 days of measurement. Thus, we partially accept our second hypothesis for DMI and ECM, but not for body temperature.

Dry matter intake was expected to be greater in the cows that were offered the high amount of forage than in those offered the low amount because the cows offered the low amount had their intake deliberately restricted. However, the cows that were offered the high amount of feed were also expected to have a greater decline in DMI during the heat challenge than those offered the low amount [36], but this was not the case in our experiment. While we did see numerical treatment differences in the decline in the DMI of our cows, these differences were not significant, illustrating a large variation in response between individual cows. During the first few days of the recovery period, there was a tendency for the cows that were offered the high amount of forage to have a greater increase in DMI than the cows offered the low amount, but this result could be confounded by us allowing those cows on the high amount to have ad libitum intake of feed instead of the previously capped amount. There was also an interaction between feed amount and feed type, but we have not been able to untangle this.

Energy-corrected milk was expected to be greater in the cows that were offered the high amount of feed than in those offered the low amount because of the greater nutrient intake. However, the difference in milk yield over the 10-day measurement period was less than the 4.4 kg predicted by the difference in ME intake (5 MJ/kg milk, [29]). This discrepancy could be due to a difference in energy partitioning during the heat challenge, which has been reported previously [37]. Untangling the response in our experiment is complicated by the interaction observed between the effect of feed type and feed amount and is thought to be driven by the cows that were offered the PS-H diet not producing more milk than those cows offered the PS-L diet. The yield of ECM was observed to increase from the pre-challenge to the heat challenge in the cows that were fed the high amount of forage, but it decreased in the cows fed the low amount. This is despite the fact that the cows that were fed the high amount of forage had a greater maximum body temperature during the heat challenge than the cows fed the low amount. This greater temperature would be expected to have a greater depressive effect on feed intake [34] in cows fed the high amount of forage compared to those offered the low amount. Our results suggest that reducing the amount of feed offered to cows during a heat challenge could result in favorable outcomes with respect to body temperature, but further work is necessary to elucidate the effect of high feed amount during heat challenge.

Body temperature was expected to be affected by the amount of feed eaten, since heat production has been positively correlated with DMI [20], and a greater heat production could be expected to result in a greater body temperature. It is possible that the cows that were offered a high amount of feed could shed heat at a greater rate than the cows offered a low amount of feed, thereby maintaining their body temperature. However, we have no evidence to support this. During the heat challenge, those cows with a greater DMI did have a greater body temperature, greater respiration rates and greater skin temperatures, which does agree with heat production being positively correlated with MEI. This correlation was established in sheep in metabolism cages [20], so the difference in observations across periods of our experiment could be due to the correlation only being apparent when our cows were under thermal stress.

Restricting the intake of our cows during a heat challenge did not affect the decline in intake from thermoneutral conditions, and there was a negative impact on milk production over all weather conditions. There was a beneficial effect of restricted intake on body temperature during the heat challenge, but we consider this single advantage to be insufficient to justify restricting feed intake as a strategy for managing hot weather events.

### 4.3. Heat Challenge

In contrast to our third hypothesis, diet type had no effect on the decrease in DMI or ECM due to the heat challenge.

The cows that were offered chicory had a greater observed numerical decline in DMI from the pre-challenge to the heat-challenge than the cows offered pasture silage, but the large variation in individual cow DMI meant that no statistical difference between the decline in DMI was identified. The low fiber concentration in chicory relative to pasture silage was expected to result in a greater cow DMI during the heat challenge period [11,12]. However, there is a report showing the decline in intake by animals with high intakes of a low-fiber diet during hot weather being greater than for those animals offered a high-fiber diet [36]. A possible explanation for our observations is gut fill. This is because the cows that were offered chicory consumed 106 kg of fresh material, while the cows offered silage only consumed 29 kg of fresh material due to the difference in dry matter of the forages (approximate DM: chicory 10%; pasture silage 35%).

The cows that were offered chicory had a lower body temperature and tended to have lower skin temperatures than those offered pasture silage. This agrees with previous reports in which cows had lower rectal temperatures when fed low NDF forage compared to high NDF forage [12]. The body temperature appears more closely related to the intake of structural carbohydrates than the proportions of individual structural carbohydrates per se, as more fibrous feeds produce greater amounts of acetate, generating significant heat in the rumen [8].

It has been suggested that the amount of feed offered during hot weather may be more critical to total metabolic heat production than the fiber concentration of the feed [10]. In this experiment, the loss in production during the heat challenge was more pronounced in the cows that were offered less feed. The change in ECM from the pre-challenge to the heat challenge period was positive for the cows that were offered the high amount of forage, being 2.39 kg ECM greater in chicory compared to pasture-silage-fed cows. In contrast, the change in ECM observed in the cows that were offered the low amount of feed was negative, with the reduction in ECM yield being 1.27 kg ECM less in the chicory-fed cows compared to those fed pasture silage. While the cows that were offered the low amount of pasture silage consumed less feed than those offered the high amount, they had similar milk yields throughout the experiment. The reason for this is unclear. A possible confounding effect is the change of environment on the day before the heat challenge, when cows were moved to the controlled-environment chambers. Any effect from the change in location was minimized by familiarizing the cows with the controlled-environment chambers before the start of the experiment. The reason for the observed increase in milk yield from the pre-challenge to the heat challenge period is not clear, but it may be due to our allocation of effects to days. We allocated milk records such that milk produced was matched to the feed consumed prior to that milking. The weather cycle, and hence the heat challenge, were synchronized with the feeding cycle. Despite this, we observed the minimum milk yield on the first day of the recovery, indicating that there was a lag effect of the heat challenge. A lag in the effect of hot weather has been recognized previously, with weather up to 4 days prior to a milking reported to affect the milk yield [32]. Given the short duration of our heat challenge and the potentially long lag effect, we conclude that it is difficult to interpret the change from the pre-challenge to the heat challenge. We recommend that our 10-day average data be used to assess the merits of chicory against those of pasture silage.

### 4.4. Blood Biochemistry

During the experiment, we measured only minor changes to the blood biochemical profiles of our cows due to each of feed type, feed amount, and exposure to the heat challenge. Before exposure to heat, blood pH tended to be greater in the cows that were fed the high amount compared to the low amount of feed, but it remained within the expected normal range (7.35 to 7.50; [38]). During the heat challenge, blood pH remained within normal limits but was greater than during the pre-challenge period. This suggests that there was a minimal disturbance of the acid–base balance of blood during the short period of thermal stress. During the pre-challenge period, blood glucose concentration tended to be lower in the cows that were fed chicory compared to pasture silage, and this trend became significant during the heat challenge. As previous research has shown that cows with greater milk yields have lower blood glucose concentrations [39], our results may indicate a greater uptake of glucose by the mammary gland of cows that are fed chicory to support the synthesis of greater milk volumes observed in this experiment. High concentrations of BHB in the blood have been correlated with oxidative stress and apoptosis in the liver [40]. The cows in this experiment had BHB concentrations well below the threshold for ketosis of 1.0 to 1.4 mmol [41] before and during the heat challenge. This indicates that the level of thermal stress induced by the heat challenge was mild. The lower concentration of BHB in the serum of the cows that were fed chicory compared to those fed pasture silage is likely explained by butyric acid in the silage being converted to blood BHB [42].

### 4.5. Feed Presentation

The manner of offering feed to our cows leaves several questions. Feeding cool feed to hot cows may mean that our results are disconnected from what would have happened had the feed experienced the same environmental conditions as the cows. The chicory was harvested from ambient conditions, but conditions were cool. The silage was collected from the stack once daily in the afternoon and the PM feed was left to sit in ambient (cool) conditions, but the AM feed was stored at 4 °C until required. There is also the fact that our feeds were offered in individual bins where there was no competition as exists when cows are grazing. Added to this is the impact that hot weather, similar to that used to challenge the cows, has on the nutritional and palatability aspects of the forage [43]. Given these deviations from conditions that could be expected on commercial farms, further research is required to validate these results under grazing scenarios where the forage is subjected to the same environmental conditions as the cows.

### 4.6. Heat Stress Risk Assessment under Experiment Conditions

The use of the HSRT in this experiment, using multiple indicators of heat stress (respiration rate, body temperature, panting score) rather than a single method (body temperature only), resulted in more animals safely completing the heat challenge period and remaining under experimental conditions. The increased frequency of monitoring triggered by a combination of respiratory and internal measures of heat stress provided a good balance between the experiment requirements and the animals’ welfare. This improved the proportion of animals that completed the heat challenge in this experiment (27 of 29 cows) compared with previous experiments (12 of 24 cows [27] and 18 of 24 cows [44]) that used a body temperature threshold of 40.9 °C as the only criterion at which cows were to be cooled before the end of the heat challenge; this improved the statistical power and efficiency of the experimental design. We recommend the use of a heat-stress risk assessment that uses multiple indicators of heat stress such as the one described in this manuscript in future controlled heat-exposure experiments.

## 5. Conclusions

Forage type affected the ECM yield and body temperature of the cows across the 10 days of measurement. The cows that were offered fresh chicory produced more milk and had a lower body temperature than those that were offered pasture silage, despite consuming similar amounts of feed. However, the type of forage offered had no impact on the change in DMI or ECM during the heat challenge. While feeding chicory may not affect the change in body temperature due to a heat challenge, the resulting lower body temperature during the heat challenge indicated that the cows that were offered chicory experienced less thermal stress than those that were offered pasture silage.

The amount of forage that was offered had no significant effect on body temperature over the 10-day observation period. However, offering more forage resulted in a greater DMI and ECM yield than when a lower amount of forage was offered. Restricting the intake of our cows during the heat challenge did not affect the decline in intake from thermoneutral conditions, and there was a negative impact on milk production over all weather conditions. There was a beneficial effect of restricted intake on body temperature during the heat challenge, but we consider this single advantage to be insufficient to justify the restriction of feed intake as a strategy for managing hot weather events.

It is important to note that our forage was not subjected to the same weather/environmental conditions as our cows and that our feeds were offered in individual bins where there was no competition—as there is when cows are grazing. There is a need to validate our current results, wherein the feed should be grazed as in a commercial farm setting and therefore subjected to the same environmental conditions as the cows.

## Figures and Tables

**Table 1:** Composition of main dietary ingredients (g/kg DM unless otherwise stated).

Parameter	Grain Mix ^1^	Chicory	Pasture Silage
Dry matter (g/kg as fed)	878	99	350
Crude protein	143	201	157
Soluble protein (% CP)	13.9	15.7	14.7
Acid detergent fiber	42	289	344
Neutral detergent fiber	93	338	479
Lignin	11	106	30
Non fiber carbohydrate	670	274	197
Starch	551	10	11
Ash	66	139	115
Total digestible nutrients	822	577	652
Calcium	15	16	6.3
Magnesium	7.4	5.4	2.0
Sodium	1.0	9.0	4.2
Potassium	4.7	33	35
Chloride	2.8	18	12
DCAD (meq./100 g DM)	−3.8	44	55
Copper (mg/kg DM)	86	8.8	13
Sulfur	1.9	4.8	3.1
Crude fat	29	48	52
ME ^2^ (MJ/kg DM)	13.9	10.1	9.2

^1^ Grain mix consisted of cracked wheat grain (815 g/kg DM), cracked lupins (112 g/kg DM), minerals (51 g/kg DM), E Mag 523 (6 g/kg DM, Queensland Magnesia Pty Ltd., Toowong, QLD, Australia), and limestone (16 g/kg DM). ^2^ ME = metabolizable energy.

**Table 2:** Blocking structure and treatment allocation to chambers for each of the 5 cohorts.

Cohort	Chamber 1	Chamber 2	Chamber 3	Chamber 4	Chamber 5	Chamber 6
1	CH-L	PS-H	CH-H	PS-L	CH-H	PS-L
2	CH-L	PS-L	PS-H	CH-H	CH-H	PS-H
3	PS-L	CH-H	PS-H	PS-H	PS-L	CH-L
4	CH-H	CH-L	PS-L	CH-H	CH-L	PS-H
5	PS-H	PS-L	CH-L	CH-L	PS-H	CH-H

**Table 3:** Calculating the heat-stress risk total of individual cows housed in controlled-climate chambers under hot conditions by rating risk of thermal stress measured by observations of panting score, respiration rate, and rectal temperature.

Panting Score ^1^	Risk Rating		Respiratory Rate ^2^	Risk Rating		Rectal Temperature ^3^	Risk Rating		
0 to 2.5	0		≤120	0		≤40.9	0		
3	1.5		121 to 159	1		41.0 to 41.5	1		
3.5	2		≥160	2		41.6 to 41.9	2		
4	3		Variable	3		≥42	3		
4.5	4								
PS risk		+	RR risk		+	RT risk		=	HSRT ^4^

^1^ PS obtained by visual observation of animals according to [25]. ^2^ RR—breaths per minute, obtained by visual observation. ^3^ RT, °C, obtained via large animal rectal thermometer. ^4^ HRST—Heat-stress risk total, calculated from the sum of risk from each of PS, RR and RT.

**Table 4:** Comparisons of the effects of pre-challenge (P), heat challenge (H) and recovery (R) periods on means of total DMI (kg/day), milk yield (kg/day), ECM yield (kg/day), milk fat concentration (g/kg), milk protein concentration (g/kg), milk lactose concentration (g/kg), vaginal temperature (°C) and respiration rate (breaths/min).

Item		Period			SED ^1^		Contrast *p*-Value ^2^		
	P	H	R	P-H	H-R	P-R	P-H	H-R	P-R
n	27	27	27						
Total DMI	15.2	14.5	15.6	0.702	0.389	0.602	0.296	0.035	0.566
Milk yield	20.0	20.1	18.7	0.412	0.335	0.466	0.598	0.013	0.064
ECM yield ^3^	19.4	19.4	18.0	0.690	0.336	0.663	0.956	0.015	0.115
Milk fat	0.77	0.78	0.71	0.041	0.016	0.039	0.864	0.013	0.194
Milk protein	0.62	0.60	0.58	0.014	0.009	0.016	0.277	0.105	0.078
Milk lactose	0.99	1.00	0.90	0.024	0.017	0.029	0.422	0.003	0.045
Vaginal temperature	38.7	39.1	38.8	0.026	0.050	0.025	<0.001	0.002	0.120
Respiration rate	32.3	78.5	44.9	3.188	4.190	2.189	<0.001	0.001	0.005

^1^ SED = standard error of difference between means for the following contrasts: pre-challenge to heat challenge (P–H); heat challenge to recovery (H–R) and pre-challenge to recovery (P–R). ^2^ The *p*-value is based on t-tests, as described in the method section. ^3^ ECM = energy corrected milk yield (kg/d).

**Table 5:** Nutrient intake (kg/d unless specified) across the 10 days of measurement and the intake of dry matter (DMI, kg/d) and metabolizable energy (MEI, MJ/d) during selected periods of the experiment.

	Main Effect ^1^									Treatment Means ^2^				
	Feed		Amount		SEDm ^3^		*p* Value							
	CH	PS	High	Low	Feed	Amount	Feed	Amount	Feed × Amount	CH-H ^4^	CH-L	PS-H	PS-L	SEDt ^5^
n	14	13	14	13						7	7	7	6	
10-day mean ^6^														
Total DMI	15.4	15.2	16.5	14.1	0.42	0.41	0.558	<0.001	0.246	16.9 ^b^	14.0 ^a^	16.1 ^b^	14.2 ^a^	0.58
CP	2.80	2.29	2.76	2.33	0.069	0.066	<0.001	<0.001	0.080	3.08 ^c^	2.52 ^b^	2.44 ^b^	2.13 ^a^	0.095
NDF	4.05	5.43	5.23	4.25	0.163	0.155	<0.001	<0.001	0.780	4.52 ^b^	3.58 ^a^	5.94 ^c^	4.92 ^b^	0.225
NFC	6.20	5.33	6.01	5.52	0.135	0.129	<0.001	0.001	0.074	6.58 ^c^	5.83 ^b^	5.45 ^ab^	5.21 ^a^	0.187
Starch	2.80	2.77	2.79	2.78	0.021	0.020	0.188	0.597	0.626	2.81	2.78	2.77	2.77	0.030
Fat	0.65	0.65	0.72	0.58	0.020	0.019	0.757	<0.001	0.660	0.72 ^b^	0.58 ^a^	0.72 ^b^	0.59 ^a^	0.028
MEI (MJ/d)	175	162	179	159	5.0	4.7	0.020	<0.001	0.149	190 ^b^	161 ^a^	169 ^a^	156 ^a^	6.9
Pre-challenge														
DMI	15.4	15.1	16.4	14.0	0.36	0.35	0.375	<0.001	0.510	16.8 ^b^	14.1 ^a^	16.1 ^b^	14.0 ^a^	0.51
MEI	176	163	179	159	4.9	4.7	0.019	<0.001	0.416	188 ^c^	163 ^ab^	171 ^b^	154 ^a^	6.8
Heat challenge														
DMI	14.2	14.7	15.4	13.5	0.69	0.67	0.420	0.008	0.420	15.4 ^b^	12.9 ^a^	15.4 ^b^	14.0 ^ab^	0.96
MEI	163	158	169	152	72	6.9	0.489	0.021	0.253	176 ^b^	150 ^a^	162 ^ab^	154 ^a^	10.0
Pre challenge to heat														
ΔDMI ^7^	−1.3	−0.3	−1.0	−0.6	0.65	0.64	0.186	0.518	0.632	−1.3	−1.2	−0.7	0.1	0.92
ΔMEI	−12.4	−4.5	−10.3	−6.6	6.44	6.15	0.259	0.574	0.498	−12.0	−12.7	−8.5	−0.4	8.90
Initial recovery														
ΔDMI ^8^	1.0	−0.4	0.8	−0.2	0.62	0.60	0.022	0.086	0.017	2.4 ^b^	−0.3 ^a^	−0.7 ^a^	−0.1 ^a^	0.87
ΔMEI	9.4	−3.6	8.2	−2.5	5.97	5.69	0.031	0.057	0.013	22.8 ^b^	−4.1 ^a^	−6.3 ^a^	−1.0 ^a^	8.24

^1^ Main effects: CH = chicory, PS = pasture silage; ^2^ treatment means comparison was performed based on Fisher’s unprotected LSD; ^3^ SEDm = standard error of the difference between main effects; ^4^ treatment diet means: CH-H = chicory high amount, CH-L = chicory low amount, PS-H = pasture silage high amount, PS-L = pasture silage low amount; ^5^ SEDt = standard error of the difference between treatments; ^6^ 10-day period = (3-day base period, 1-day pre-challenge period, 2-day heat challenge period and 4-day recovery period); ^7^ Δ variable = (heat challenge variable—pre-challenge variable); ^8^ Δ variable = (recovery day 2 variable—recovery day 1 variable); ^a,b^ treatment means with different superscripts are different (*p* < 0.05).

**Table 6:** Milk yield and yield of milk components (kg/d) and milk composition (g/kg) across the 10 days of measurement and during selected periods of the experiment.

	Main Effect ^1^									Treatment Means ^2^				
	Feed		Amount		SEDm ^3^		*p* Value							
	CH	PS	High	Low	Feed	Amount	Feed	Amount	Feed × Amount	CH-H ^4^	CH-L	PS-H	PS-L	SEDt ^5^
n	14	13	14	13						7	7	7	6	
10-day mean ^6^														
Milk yield	21.9	17.2	20.5	18.7	0.55	0.53	<0.001	0.003	0.003	23.7 ^c^	20 ^b^	17.2 ^a^	17.3 ^a^	0.76
ECM yield ^7^	20.7	17.1	20.0	17.9	0.29	0.28	<0.001	<0.001	<0.001	22.6 ^c^	18.9 ^b^	17.3 ^a^	16.9 ^a^	0.41
Fat yield	0.78	0.72	0.79	0.71	0.024	0.023	0.017	0.002	0.035	0.85 ^b^	0.72 ^a^	0.73 ^a^	0.71 ^a^	0.033
Protein yield	0.71	0.50	0.65	0.56	0.022	0.022	<0.001	0.001	0.021	0.78 ^c^	0.63 ^b^	0.52 ^a^	0.48 ^a^	0.031
Fat concentration	36	43	39	39	1.5	1.6	<0.001	0.916	0.363	35 ^a^	36 ^a^	43 ^b^	42 ^b^	2.2
Protein concentration	32	29	32	30	0.7	0.7	0.001	0.055	0.353	33 ^c^	32 ^bc^	31 ^ab^	28 ^a^	1.0
Pre-challenge														
Milk yield	21.8	18.1	19.9	20.0	1.07	1.04	0.002	0.925	0.038	22.9 ^c^	20.7 ^bc^	16.8 ^a^	19.3 ^ab^	1.49
ECM yield	20.7	18.1	19.3	19.5	1.09	1.05	0.021	0.849	0.099	21.5 ^b^	19.9 ^ab^	17.0 ^a^	19.2 ^ab^	1.52
Fat yield	0.78	0.77	0.75	0.79	0.060	0.057	0.761	0.554	0.397	0.79	0.77	0.72	0.81	0.083
Protein yield	0.71	0.53	0.64	0.61	0.037	0.036	<0.001	0.356	0.101	0.76 ^b^	0.66 ^b^	0.52 ^a^	0.55 ^a^	0.051
Fat concentration	35	43	39	40	2.2	2.2	0.003	0.516	0.254	34 ^a^	38 ^ab^	44 ^b^	43 ^b^	3.1
Protein concentration	33	30	32	30	0.91	0.88	0.017	0.062	0.270	33 ^b^	32 ^b^	31 ^b^	29 ^a^	1.3
Heat challenge														
Milk yield	22.7	17.4	21.2	19.0	0.75	0.73	<0.001	0.006	0.014	24.9 ^c^	20.6 ^b^	17.5 ^a^	17.3 ^a^	1.05
ECM yield	21.6	17.1	20.7	18.0	0.58	0.56	<0.001	<0.001	<0.001	24.2 ^c^	19 ^b^	17.2 ^a^	17.0 ^a^	0.80
Fat yield	0.83	0.73	0.83	0.73	0.033	0.032	0.005	0.004	0.003	0.93 ^b^	0.72 ^a^	0.72 ^a^	0.74 ^a^	0.046
Protein yield	0.72	0.49	0.66	0.55	0.027	0.026	<0.001	0.001	0.057	0.8 ^c^	0.63 ^b^	0.51 ^a^	0.46 ^a^	0.038
Fat concentration	36	42	40	39	1.6	1.6	0.001	0.701	0.454	37 ^a^	35 ^a^	42 ^b^	43 ^b^	2.2
Protein concentration	31	28	31	29	0.9	0.8	0.004	0.025	0.220	32 ^b^	31 ^b^	30 ^b^	27 ^a^	1.2
Pre challenge to heat														
∆Milk yield ^8^	1.0	−0.7	1.3	−1.1	1.08	1.05	0.165	0.035	0.754	1.98 ^b^	−0.08 ^ab^	0.68 ^ab^	−2.05 ^a^	1.497
∆ECM yield	0.9	−1.0	1.4	−1.5	1.25	1.20	0.180	0.026	0.659	2.61 ^b^	−0.85 ^ab^	0.22 ^ab^	−2.12 ^a^	1.737
∆Fat yield	0.05	−0.04	0.07	−0.06	0.064	0.061	0.184	0.035	0.318	0.15 ^b^	−0.05 ^a^	−0.01 ^ab^	−0.07 ^a^	0.088
∆Protein yield	0.00	−0.04	0.02	−0.06	0.034	0.033	0.214	0.032	0.794	0.04 ^b^	−0.03 ^ab^	0.00 ^ab^	−0.09 ^a^	0.047
∆Fat concentration	0.5	−0.8	0.9	−1.2	1.82	1.86	0.413	0.278	0.055	3.4 ^b^	−2.3 ^a^	−1.7 ^ab^	0.0 ^ab^	2.60
∆Protein concentration	−1.2	−1.7	−1.3	−1.6	0.31	0.30	0.148	0.345	0.838	−1.1	−1.3	−1.5	−1.9	0.44
Initial recovery														
∆Milk yield ^9^	1.2	0.6	0.8	1.0	0.51	0.50	0.212	0.746	0.120	1.5	0.87	0.1	1.11	0.71
∆ECM yield	1.0	0.6	0.5	1.1	0.67	0.64	0.471	0.400	0.081	1.4	0.7	−0.28	1.55	0.931
∆Fat yield	0.01	0.01	−0.01	0.04	0.044	0.040	0.929	0.268	0.143	0.02	0.01	−0.05	0.08	0.061
∆Protein yield	0.07	0.04	0.05	0.06	0.014	0.014	0.046	0.671	0.100	0.08 ^b^	0.06 ^ab^	0.03 ^a^	0.06 ^ab^	0.020
∆Fat concentration	−1.1	−0.4	−1.4	−0.0	2.39	2.43	0.842	0.566	0.393	−0.8	−1.4	−2.1	1.4	3.39
∆Protein concentration	1.7	1.7	1.6	1.8	0.35	0.33	0.910	0.522	0.948	1.6	1.8	1.6	1.8	0.48

^1^ Main effects: CH = chicory, PS = pasture silage; ^2^ treatment means comparison was performed based on Fisher’s unprotected LSD; ^3^ SEDm = standard error of the difference between main effects; ^4^ treatment diet effects: CH-H = chicory high amount, CH-L = chicory low amount, PS-H = pasture silage high amount, PS-L = pasture silage low amount; ^5^ SEDt = standard error of the difference between treatments; ^6^ 10-day period = (3-day base period, 1-day pre-challenge period, 2-day heat challenge period and 4-day recovery period); ^7^ ECM = energy-corrected milk yield (kg/day); ^8^ Δ variable = (heat challenge variable—pre-challenge variable); ^9^ Δ variable = (recovery day 2 variable—recovery day 1 variable); ^a,b^ treatment means with different superscripts are different (*p* < 0.05).

**Table 7:** Daily mean and maximum vaginal temperature (VT; °C) and duration of vaginal temperature greater than 38.8 °C (min/day) across the 10 days of measurement and during selected periods of the experiment.

	Main Effect ^1^									Treatment Means ^2^				
	Feed		Amount		SEDm ^3^		*p* Value							
	CH	PS	High	Low	Feed	Amount	Feed	Amount	Feed × Amount	CH-H ^4^	CH-L	PS-H	PS-L	SEDt ^5^
n	14	13	14	13						7	7	7	6	
10-day mean ^6^														
Mean VT	38.8	38.9	38.8	38.8	0.03	0.03	0.022	0.306	0.994	38.8 ^ab^	38.8 ^a^	38.9 ^b^	38.8 ^ab^	0.05
Maximum VT	39.4	39.6	39.5	39.5	0.05	0.05	0.024	0.718	0.626	39.4 ^a^	39.5 ^ab^	39.6 ^b^	39.5 ^ab^	0.07
Duration > 38.8 °C	569	687	646	609	67.1	61.9	0.095	0.556	0.832	580	557	712	661	90.8
Pre-challenge														
Mean	38.6	38.8	38.7	38.7	0.04	0.04	0.001	0.825	0.850	38.6 ^a^	38.6 ^a^	38.8 ^b^	38.8 ^b^	0.06
Maximum	39.2	39.3	39.2	39.3	0.05	0.05	0.005	0.317	0.045	39.1 ^a^	39.2 ^b^	39.4 ^b^	39.3 ^b^	0.07
Duration > 38.8 °C	312	634	468	478	99.7	91.9	0.005	0.915	0.974	306 ^a^	319 ^a^	631 ^b^	637 ^b^	134.8
Heat challenge														
Mean	39.1	39.3	39.3	39.1	0.10	0.09	0.048	0.059	0.705	39.1 ^ab^	39 ^a^	39.4 ^b^	39.1 ^ab^	0.14
Maximum	39.8	40.1	40.1	39.7	0.14	0.14	0.059	0.011	0.972	40.0 ^ab^	39.6 ^a^	40.3 ^b^	39.9 ^ab^	0.20
Duration > 38.8 °C	914	1233	1128	1020	110	101	0.010	0.303	0.954	965 ^a^	863 ^a^	1290 ^b^	1176 ^ab^	148.7
Pre challenge to heat														
ΔMean ^7^	0.42	0.46	0.53	0.35	0.099	0.094	0.608	0.069	0.763	0.49	0.34	0.57	0.36	0.138
ΔMaximum	0.63	0.73	0.90	0.46	0.148	0.141	0.448	0.006	0.428	0.91 ^b^	0.35 ^a^	0.89 ^b^	0.58 ^ab^	0.207
ΔDuration > 38.8 °C	602	599	660	542	115	106	0.984	0.282	0.978	659	544	660	539	155.1
Initial recovery														
ΔMean ^8^	0.09	0.16	0.15	0.10	0.041	0.039	0.085	0.183	0.961	0.12 ^ab^	0.06 ^a^	0.19 ^b^	0.14 ^ab^	0.056
ΔMaximum	0.14	0.21	0.22	0.13	0.075	0.071	0.382	0.170	0.046	0.27 ^b^	0.01 ^a^	0.18 ^ab^	0.25 ^b^	0.104
ΔDuration > 38.8 °C	126	194	150	170	114	105	0.566	0.853	0.895	123	129	176	211	154.1

^1^ Main effects: CH = chicory, PS = pasture silage; ^2^ treatment means comparison was performed based on Fisher’s unprotected LSD; ^3^ SEDm = standard error of the difference between main effects; ^4^ treatment diet effects: CH-H = chicory high amount, CH-L = chicory low amount, PS-H = pasture silage high amount, PS-L = pasture silage low amount; ^5^ SEDt = standard error of the difference between treatments; ^6^ 10-day period = (3-day base period, 1-day pre-challenge period, 2-day heat challenge period and 4-day recovery period); ^7^ Δ variable = (heat challenge variable—pre-challenge variable); ^8^ Δ variable = (recovery day 2 variable—recovery day 1 variable); ^a,b^ treatment means with different superscripts are different (*p* < 0.05).

**Table 8:** Respiration rate (breaths per min) and skin temperatures (°C) at 14:45 h during selected periods of the experiment.

	Main Effect ^1^									Treatment Means ^2^				
	Feed		Amount		SEDm ^3^		*p* Value							
	CH	PS	High	Low	Feed	Amount	Feed	Amount	Feed × Amount	CH-H ^4^	CH-L	PS-H	PS-L	SEDt ^5^
n	14	13	14	13						7	7	7	6	
Pre-challenge														
Respiration rate	35	30	32	33	2.6	2.5	0.063	0.812	0.972	35	35	29	30	3.6
Left flank	30.7	31.3	31.1	30.9	0.37	0.35	0.092	0.605	0.936	30.8	30.6	31.4	31.2	0.50
Neck	30.0	30.3	30.4	29.9	0.33	0.31	0.134	0.106	0.055	29.9 ^a^	30.0 ^a^	31.0 ^b^	29.7 ^a^	0.44
Leg upper	28.1	28.3	28.7	27.6	0.24	0.23	0.086	<0.001	0.004	28.2 ^b^	27.9 ^ab^	29.2 ^c^	27.3 ^a^	0.32
Leg lower	26.8	27.4	27.8	26.4	0.52	0.49	0.178	0.014	0.740	27.6 ^b^	26.1 ^a^	28.0 ^b^	26.8 ^ab^	0.70
Heat challenge														
Respiration rate	85	72	87	70	7.4	7.0	0.117	0.035	0.776	92 ^b^	78 ^ab^	81 ^ab^	63 ^a^	10.2
Left flank	34.8	35.0	35.2	34.6	0.25	0.24	0.411	0.015	0.443	35.2 ^b^	34.4 ^a^	35.2 ^b^	34.8 ^ab^	0.35
Neck	34.9	34.8	35.2	34.5	0.27	0.26	0.633	0.008	0.607	35.4 ^b^	34.5 ^a^	35.1 ^ab^	34.5 ^a^	0.38
Leg upper	33.2	33.5	33.8	33.0	0.19	0.18	0.097	0.001	0.938	33.6 ^bc^	32.9 ^a^	33.9 ^c^	33.2 ^ab^	0.27
Leg lower	33.5	33.8	34.0	33.2	0.23	0.22	0.265	0.001	0.553	34 ^bc^	33 ^a^	34.1 ^c^	33.4 ^ab^	0.31

^1^ Main effects: CH = chicory, PS = pasture silage; ^2^ treatment means comparison was performed based on Fisher’s unprotected LSD; ^3^ SEDm = standard error of the difference between main effects; ^4^ treatment diet effects: CH-H = chicory high amount, CH-L = chicory low amount, PS-H = pasture silage high amount, PS-L = pasture silage low amount; ^5^ SEDt = standard error of the difference between treatments; ^a,b,c^ treatment means with different superscripts are different (*p* < 0.05).

**Table 9:** Selected blood parameters during selected periods of the experiment. Blood pH and serum concentrations of beta-hydroxy butyrate (BHB; mmol/L), non-esterified fatty acids (NEFA; mmol/L), glucose (mmol/L), haptoglobin (g/L), Na+ (mmol/L), and K+ (mmol/L).

	Main Effect ^1^									Treatment Means ^2^				
	Feed		Amount		SEDm ^3^		*p* Value							
	CH	PS	High	Low	Feed	Amount	Feed	Amount	Feed × Amount	CH-H ^4^	CH-L	PS-H	PS-L	SEDt ^5^
n	14	13	14	13						7	7	7	6	
Pre-challenge														
pH	7.41	7.44	7.45	7.40	0.023	0.022	0.173	0.069	0.519	7.43 ^ab^	7.40 ^a^	7.47 ^b^	7.41 ^ab^	0.032
BHB	0.59	0.65	0.61	0.63	0.061	0.060	0.292	0.684	0.456	0.55	0.62	0.66	0.64	0.086
NEFA	0.09	0.11	0.10	0.11	0.015	0.015	0.160	0.280	0.541	0.09	0.10	0.10	0.13	0.022
Glucose	3.0	3.3	3.2	3.1	0.11	0.10	0.062	0.328	0.721	3.1 ^ab^	3.0 ^a^	3.3 ^ab^	3.2 ^b^	0.15
Haptoglobin	0.21	0.22	0.22	0.22	0.008	0.008	0.301	0.968	0.435	0.21	0.22	0.23	0.22	0.012
Na	139	139	140	139	1.1	1.1	0.619	0.358	0.882	139	138	140	139	1.5
K	4.4	4.6	4.6	4.4	0.19	0.19	0.256	0.302	0.350	4.5	4.2	4.6	4.6	0.27
Heat challenge (H2)														
pH	7.45	7.46	7.46	7.46	0.020	0.019	0.531	0.758	0.053	7.43	7.48	7.48	7.45	0.03
BHB	0.47	0.59	0.53	0.53	0.041	0.040	0.006	0.975	0.271	0.45 ^a^	0.49 ^ab^	0.62 ^c^	0.57 ^bc^	0.057
NEFA	0.08	0.09	0.07	0.10	0.014	0.014	0.612	0.159	0.091	0.08 ^ab^	0.08 ^ab^	0.07 ^a^	0.11 ^b^	0.020
Glucose	3.2	3.4	3.3	3.3	0.105	0.102	0.024	0.571	0.406	3.2 ^ab^	3.1 ^a^	3.4 ^b^	3.4 ^b^	0.15
Haptoglobin	0.24	0.22	0.22	0.24	0.011	0.010	0.248	0.090	0.855	0.23 ^ab^	0.24 ^b^	0.21 ^a^	0.23 ^ab^	0.015
Na	140	141	141	140	0.593	0.566	0.296	0.119	0.413	141	140	142	140	0.82
K	4.8	4.8	4.1	4.8	0.179	0.171	0.760	0.953	0.777	4.9	4.8	4.8	4.8	0.25

^1^ Main effects: CH = chicory, PS = pasture silage; ^2^ treatment means comparison was performed based on Fisher’s unprotected LSD; ^3^ SEDm = standard error of the difference between main effects; ^4^ treatment diet effects: CH-H = chicory high amount, CH-L = chicory low amount, PS-H = pasture silage high amount, PS-L = pasture silage low amount; ^5^ SEDt = standard error of the difference between treatments; ^a,b,c^ treatment means with different superscripts are different (*p* < 0.05).

## Data Availability

The Data are available from the corresponding author upon reasonable request.

## Supplementary Materials

The following supporting information can be downloaded at: https://www.mdpi.com/article/10.3390/ani13050867/s1, Table S1: Blood parameters.
